# Object-stable unsupervised dual contrastive learning image-to-image translation with query-selected attention and convolutional block attention module

**DOI:** 10.1371/journal.pone.0293885

**Published:** 2023-11-06

**Authors:** Yunseok Oh, Seonhye Oh, Sangwoo Noh, Hangyu Kim, Hyeon Seo

**Affiliations:** 1 Department of AI Convergence Engineering, Gyeongsang National University, Jinju-si, Gyeongsangnam-do, Republic of Korea; 2 Precedent Study Team for C4ISR Systems, Korea Research Institute for Defense Technology Planning and Advancement, Jinju-si, Gyeongsangnam-do, Republic of Korea; 3 Guided & Firepower Systems Technology Planning Team, Korea Research Institute for Defense Technology Planning and Advancement, Jinju-si, Gyeongsangnam-do, Republic of Korea; 4 C4ISR Systems Technology Planning Team, Korea Research Institute for Defense Technology Planning and Advancement, Jinju-si, Gyeongsangnam-do, Republic of Korea; 5 Clova Speech, NAVER Cloud, Seongnam-si, Gyeonggi-do, Republic of Korea; 6 Department of Computer Science, Gyeongsang National University, Jinju-si, Gyeongsangnam-do, Republic of Korea; University of California Los Angeles, UNITED STATES

## Abstract

Recently, contrastive learning has gained popularity in the field of unsupervised image-to-image (I2I) translation. In a previous study, a query-selected attention (QS-Attn) module, which employed an attention matrix with a probability distribution, was used to maximize the mutual information between the source and translated images. This module selected significant queries using an entropy metric computed from the attention matrix. However, it often selected many queries with equal significance measures, leading to an excessive focus on the background. In this study, we proposed a dual-learning framework with QS-Attn and convolutional block attention module (CBAM) called object-stable dual contrastive learning generative adversarial network (OS-DCLGAN). In this paper, we utilize a CBAM, which learns what and where to emphasize or suppress, thereby refining intermediate features effectively. This CBAM was integrated before the QS-Attn module to capture significant domain information for I2I translation tasks. The proposed framework outperformed recently introduced approaches in various I2I translation tasks, showing its effectiveness and versatility. The code is available at https://github.com/RedPotatoChip/OSUDL

## 1. Introduction

Image-to-Image (I2I) translation is a field in computer vision that aims to produce images from a source domain to a target domain while preserving essential content. The emergence of generative adversarial networks (GANs) [1] has led to great improvements in various I2I translation [2] tasks such as the translation of images of horses to zebras, low-resolution to high-resolution images, [3], and aerial photos to maps [4].

Generally, I2I translations can be categorized into paired (supervised) [4–6] and unpaired (unsupervised) task [2, 7–10]. Using paired training data, I2I translation models have shown impressive results with conditional GANs [11]. However, it is difficult and expensive to collect paired data with pixel-to-pixel mapping for training, which restricts the applicability of such methods to existing datasets and domains [4]. Unsupervised I2I translation conducts a cross-domain transfer without paired data, which is close to real-world scenarios. The main problem encountered by GANs in unsupervised I2I translation is that the adversarial loss [1] is underconstrained and there are multiple possible mappings between domains, leading to translated images with poor quality [12].

To address these limitations, adversarial loss [1] can be employed to enforce the target appearance and cycle consistency [2] to maintain content; this can be an overly restrictive approach. The cycle consistency assumption adopted by models such as CycleGAN [2], DiscoGAN [7], and DualGAN [8] can limit their ability to perform changes in geometry and forces the relationship between two domains to be a bijection [13], which is not always ideal. Furthermore, the training cost associated with these methods is higher since two generators and two discriminators are used.

**Fig 1:**
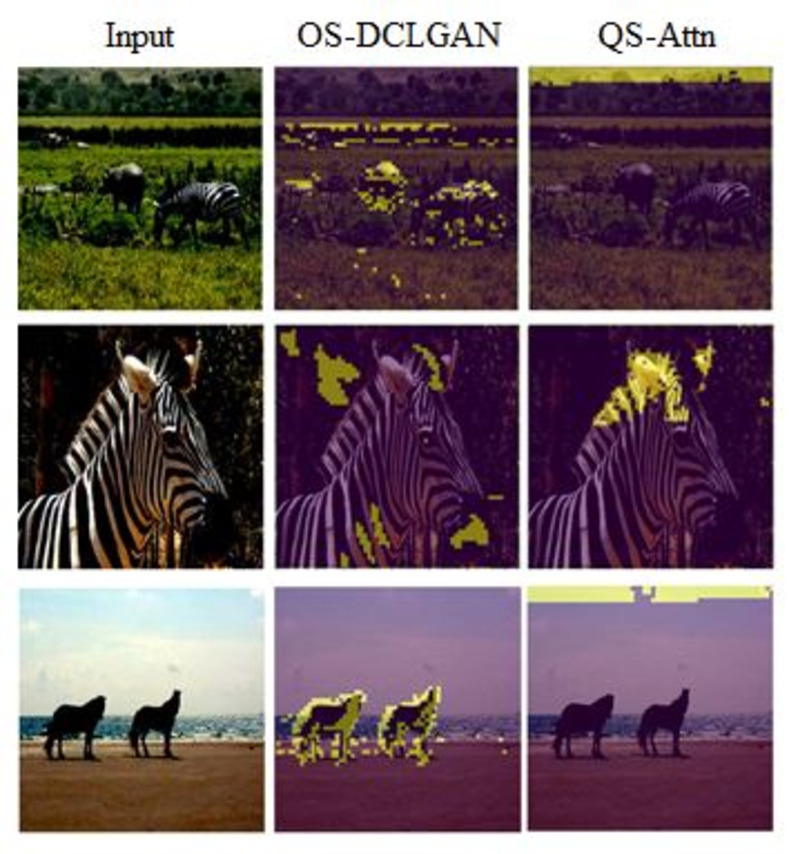
Differences in patch acquisition. The locations of 256 patches obtained proposed model (OS-DCLGAN) and QS-Attn only. The yellow areas on the right show the locations of the patches obtained.

Contrastive learning approaches that use multiple views of data have achieved state-of-the-art performances in the field of self-supervised representation learning [14–18]. Contrastive unpaired translation (CUT) [19] incorporates contrastive learning with a single embedding to maximize the mutual information between input and output image patches. Recent I2I translation methods such as the dual contrastive learning generative adversarial network (DCLGAN) [12] and query-selected attention (QS-Attn) [20] module have attempted to improve its performance. Despite the superior performance of these techniques, we found that they could not effectively capture objects corresponding to the background. When QS-Attn obtains anchor features, the entropy distribution is calculated as a metric to measure the importance of the features in reflecting the domain characteristics. Then, the smallest N points on the image are selected by sorting the entropy. We analyzed this entropy metric, finding that zero entropy points exceeded the number of samples, which resulted in important feature selections being missed. As shown in Fig 1, zero-entropy points are concentrated at the tops of the images, corresponding more to the background than the zebras.

Motivated by recent methods [12, 20], we propose an I2I translation model adapted from DCLGAN and QS-Attn called object-stable-DCLGAN (OS-DCLGAN). In particular, we modified the QS-Attn module by inserting a convolutional block attention module (CBAM) [21] to improve the stable feature representation of objects. This module was introduced for adaptive feature refinement with channel attention and spatial attention modules that supported a more efficient extraction of contextual information. The feature refinement process of CBAM enabled the QS-Attn module to obtain significant positive, negative, and anchor features.

In summary, the main contributions of this paper are as follows:

- We propose a new framework called OS-DCLGAN for stable I2I object translation, which can focus on important features. We enhanced attention module by adding CBAM blocks and show that its incorporation in QS-Attn improves the results.
- To achieve further improvement, we employ an existing state-of-the-art architecture of dual setting called DCLGAN, which enables excellent training stability.
- We present extensive experiments conducted to verify the performance of our model. The qualitative and quantitative results obtained on the benchmark datasets show that proposed method outperforms the existing state-of-the-art methods in I2I translation tasks.

## 2. Background and related work

### 2.1. Image-to-image translation

An I2I translation task refers to transforming an image from one domain to another. GANs [1] have achieved success in these tasks due to their ability to model the high-dimensional distribution of images through the adversarial loss, which attempts to make the generated image indistinguishable from the real image. As previously mentioned, I2I translation is categorized as either paired (supervised) or unpaired (unsupervised). In the paired setting [4–6], each image in the source domain has a corresponding image in the target domain. Pix2pix [4], a supervised I2I method, uses a conditional GAN(CGAN) [11] to learn the mapping function between input and output images. Regarding CGANs, they utilize supplementary details such as classification labels [22–24] or text descriptions [25–28] to steer the process of image generation. This approach results in the formation of semantic images that fulfill specific requirements. Being supervised approaches, both pix2pix and pix2pixHD [5] need paired data and the adversarial loss from a target domain discriminator for training. However, the requirement of paired data during training can pose a challenge in real-world scenarios where collecting such data is difficult. To overcome this, unpaired I2I methods [2, 7, 8, 19, 29–32] have been proposed. Unpaired setting is often achieved through cycle-consistency [2], which involves learning an inverse mapping from the output domain back to the input and ensuring the reconstruction of the input. CycleGAN [2], DiscoGAN [7] and DualGAN [8] are examples of methods that achieve I2I translation based on unpaired data by simultaneously training two generators and using cycle-consistency. This idea has been extended by methods such as unsupervised image-to-image translation (UNIT) [10] and multimodal unsupervised image-to-image translation (MUNIT) [29], which propose learning a shared intermediate “content” latent space. Diverse image-to-image translation (DRIT) [33, 34] preserves the source and target domain information during I2I translation by utilizing two discriminators. StarGAN [9, 31] employs a single discriminator to recognize the target domain while the generator tries to translate an image to multiple target domains. The pixel2style2pixel framework [35] combines the content of an image with the style of another image to generate a new image that has both content and style information. There have been recent efforts to enhance the quality of results by addressing challenges in multi-domain and multimodal synthesis [9, 36, 37]. In addition, attempts have been made to create more realistic images using the attention mechanism [38–42]. However, the assumption of cycle-consistency can sometimes be overly restrictive. Council-GAN [43] uses multiple generators and discriminators to reduce cycle-consistency constraints; however, the strong constraint on pixels can still impact image quality. To tackle this, some methods use feature-level perceptual loss or a foreground mask to guide the generator [40, 44] at the cost of increased model complexity. Both CUT [19] and F-LSeSim [45] incorporate the self-supervised contrastive loss into I2I translation, which significantly increases the translation quality. Our proposed framework, an effective two-sided I2I translation framework based on the concept of contrastive learning, is inspired by this approach.

### 2.2. Contrastive learning

Contrastive learning is a field of unsupervised learning that aims to support a model in gathering similar sample pairs while separating dissimilar pairs, enabling it to capture the valuable features or representations of the underlying structures and patterns present in data [15]. It has applications in various domains, including but not limited to image classification and style translation [46, 47]. Recently, there has been a growing trend of incorporating contrastive learning into graph domains [48, 49]. This is achieved by maximizing the mutual information between the input and generated images. Furthermore, it is necessary to align closely related patches at specific positions in the input and output images. The CUT [19] method uses noise-contrastive estimation to learn the relationship between the input image patches and corresponding generated image patches, resulting in a better performance. Another approach, DCLGAN [12], improves upon CUT by employing separate encoders and projection heads for each domain and using dual learning to effectively bridge the domain gap and stabilize the training. Then, the QS-Attn [20] module, which routes features in both domains while maintaining source relations in the synthesis, selects relevant anchor points for contrastive learning, improving the performance of CUT. In this study, we improved the performance of QS-Attn by adding channel attention, using it in a dual mode to increase training stability and maximize mutual information. The proposed model effectively extracted meaningful 256 features using the max-pooling of CBAM, which reduced the computational cost.

## 3. Methods

**Fig 2:**
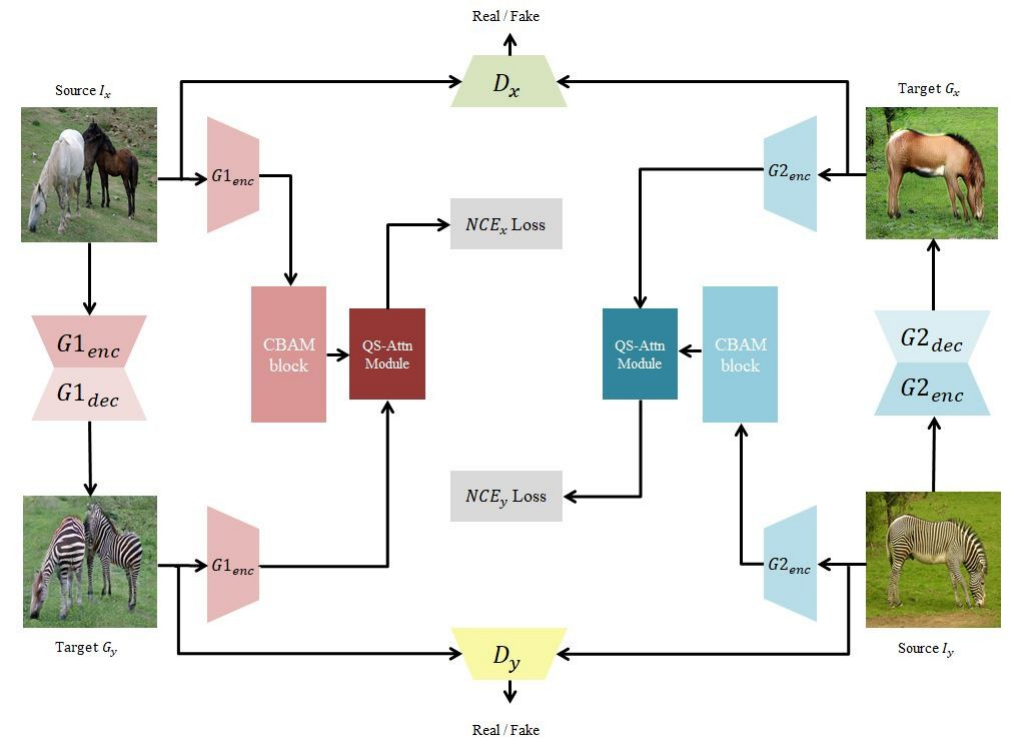
Overall structure of the proposed model. The model learns dual mappings, G1: X→Y and G2: Y→X, through dual learning, enabling I2I translation between unpaired image pairs. The CBAM and QS-Attn modules are used to selectively extract 256 patches, and the PatchNCE loss is used to make the selected patches both resemble real images and be distinguishable from fake images. Through this process, the model can perform high-quality image transformations.

**Fig 3:**
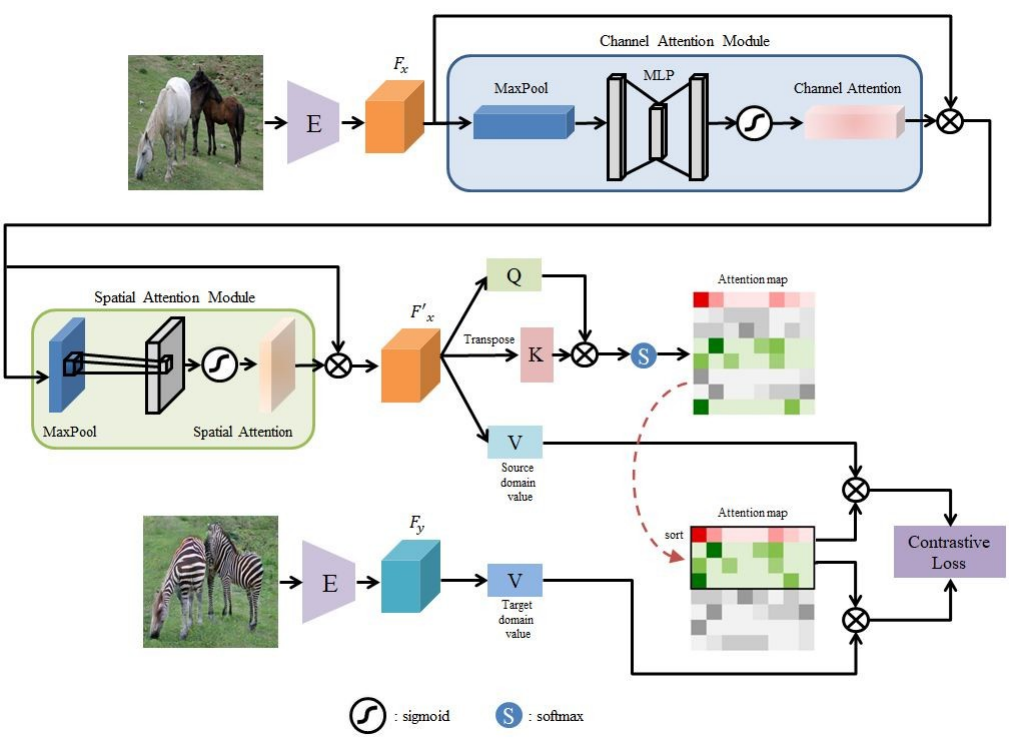
Detailed working of the proposed model. The embedding process of the model involves using the encoder E to extract features F_x_ and F_y_ from the source and target images, respectively. Using the CBAM module, the feature F_x_ of the source image is transformed into ${\text{F}}^{\prime }x$, which indicates where the focus of the input image should be. Then, the QS-Attn module is used to sort the attention matrix based on the entropy and obtain the final attention matrix with the selected N rows. The red and green patches represent the positive and negative features, respectively.

The proposed method is designed to translate images from the source domain *X* ⊂ ℝ ^H × W × C^ into images that resemble those from the target domain *Y* ⊂ ℝ ^H × W × 3^. First, a dataset of unpaired samples from both domains X = {*x* ∈ *X*} and Y = {*y* ∈ *Y*} is obtained. Two transformation functions, with G_1*x*→*y*_ converting images from X to Y and G_2*y*→*x*_ converting images from Y to X, are learnt, Two discriminators, D_x_ and D_y_, are employed to validate whether the translated images have been properly aligned with the correct image domain. Owing to the dual-mode setting of the proposed approach, there are two separate encoders and decoders from the two generators, with the first and second halves being defined as the encoder and decoder, respectively. Then, CBAM [21] is applied to the features extracted by the encoder, enabling the efficient extraction of contextual information from the images. The refined features are passed through the QS-Attn module and sent to a two-layer multilayer perceptron (MLP) projection head (H_x_ and H_y_). Regarding the attention mechanism, it involves comparing a query with the keys and then selecting the query based on the comparison outcome. Fig 2 illustrates the overall structure of the proposed model, with Fig 3 showing its detailed working.

### 3.1. Adversarial loss

The adversarial loss [1] is utilized to push the generator to produce an output that resembles the images from the target domain. In the study, we used two GANs and calculated their loss functions as follows:

$$L_{GAN}\left(G1,D_{y},X,Y\right)=\;E_{y∼Y}\left[\log D_{y}\left(y\right)\right]+E_{x∼X}\;\left[\log\left(1-D_{y}\left(G\left(x\right)\right)\right)\right]$$ (1)

$$L_{GAN}\left(G2,D_{x},X,Y\right)=\;E_{x∼X}\;\left[\log D_{x}\left(x\right)\right]+E_{y∼Y}\;\left[\log\left(1-D_{x}\left(G\left(y\right)\right)\right)\right]$$ (2)

### 3.2. Patch-based multi-layer contrastive learning

#### 3.2.1. Contrastive loss

We aimed to improve the mutual information between an input and the output by employing a noise–contrast estimation framework [17]. To capture the semantic similarities in the input space, a function that maps the input images to feature representations in the feature space, denoted as z, is learnt. This function is optimized using a contrastive loss, which encourages the proximity of feature representations z and their respective positive sample k^+^ in the feature space while simultaneously driving apart the representations of other negative pairs. The query, positive, and N negatives are denoted as *k*, *k*^+^ ∈ *R*^*K*^ and k^-^ ∈ *R*^*N*×*K*^, respectively. The application of the Euclidean norm to these vectors allows for the creation of an (N + 1)-class classification problem, where the probability of choosing the positive is calculated. Specifically, the contrastive loss is established as follows:

$$l\left(k,k^{+},k^{-}\right)=-log\frac{e^{s\left(k,k^{+}\right)/\tau }}{e^{s\left(k,k^{+}\right)/\tau }+\sum_{n}e^{s\left(k,k^{-}\right)/\tau }}$$ (3)

where τ is the temperature parameter (default value of 0.07), which was employed to scale the distance between the query vector and other examples, and s(·) is the cosine similarity.

#### 3.2.2. PatchNCE loss

We aim to match the corresponding patches of the input and output images by leveraging other patches within the input as negatives. L layers are selected from G1_enc_(X) and sent to H_x_, embedding one image into a stack of features, ${\left\{{\text{z}}_{l}\right\}}_{\text{L}}={\left\{{\text{H}}_{\text{X}}^{l}\left(\text{G}1_{\text{enc}}^{l}\left(\text{x}\right)\right)\right\}}_{L}$ where $\text{G}1_{\text{enc}}^{l}$ represents the output of the *l*-th selected layer. In a stack of features, each feature corresponds to a specific patch in an image, enabling the patch-based nature of the features to be leveraged. S_*l*_ represents the total spatial locations of each layer and is assigned using the notation. At each iteration, a query is chosen, and the corresponding feature is considered the “positive” and represented as ${\text{z}}_{i}^{\text{s}}\in {\mathbb{R}}^{C_{i}}$ while all the other features are considered “negatives” and represented as ${\text{Z}}_{l}^{S/s}\in {\mathbb{R}}^{\left(S_{i}-1\right)\times C_{i}}$, where *C*_*i*_ indicate the channel count for each layer. Similarly, another stack of features ${\left\{{\hat{z}}_{l}\right\}}_{\text{L}}={\left\{{\text{H}}_{\text{Y}}^{\text{l}}\left(\text{G}2_{\text{enc}}^{\text{l}}\left(\text{x}\right)\right)\right\}}_{L}$ is obtained. The multi-layer patch NCE loss, which maps G1: X → Y, can be represented as follows:

$$L_{PN}^{\left(X\right)}\left(G1,H_{x},H_{y},X\right)=E_{x∼X}\sum_{l}^{L}\sum_{s}^{S_{l}}l\left({\hat{z}}_{l}^{s},z_{l}^{s},z_{l}^{S\backslash s}\right)$$ (4)

A similar loss can also be introduced for the reverse mapping G2: Y → X.


$$L_{PN}^{\left(Y\right)}\left(G2,H_{x},H_{y},Y\right)=E_{y∼Y}\sum_{l}^{L}\sum_{s}^{S_{l}}l\left({\hat{z}}_{l}^{s},z_{l}^{s},z_{l}^{S\backslash s}\right)$$ (5)

### 3.3. Identity loss

To maintain the integrity of the outputs of the generator, an identity loss is implemented as follows [2]:

$$L_{identity}\left(G1,G2\right)=E_{x∼X}\left[{\left\|G2\left(G1\left(x\right)\right)-x\right\|}_{1}\right]+E_{y∼Y}\left[{\left\|G1\left(G2\left(y\right)\right)-y\right\|}_{1}\right]$$ (6)

This helps to ensure consistency in the color compositions between the input and output.

### 3.4. CBAM

The CBAM module [21] emphasizes the meaningful features along the two principal dimensions of the channel and spatial axes. Regarding its working, the channel and spatial modules are placed sequentially, and the CBAM effectively refines the intermediate features by learning the type of information that is highly relevant in terms of both content and location.

#### 3.4.1. Channel attention

Regarding the channel attention process in the CBAM, each channel of the feature map extracts meaningful features from provided input data. A channel attention map is produced to model the interdependencies between the channels and emphasize the interdependent feature maps, improving the feature representations of specific semantics. In this study, only max-pooled features were used to extract features that gathered valuable information regarding distinct object features. Channel attention can be computed as follows:

$$M_{c}\left(F\right)=\sigma \left(MLP\left(MaxPool\left(F\right)\right)\right)$$ (7)

#### 3.4.2. Spatial attention

The spatial attention mechanism differs from the channel attention mechanism in that it focuses on the location of important information instead of its meaning in the feature map. It encodes a wider range of contextual information into local features to enhance the representational capabilities. For instance, when predicting an object in an image, only the regions containing the object are useful. Therefore, the spatial attention mechanism prioritizes semantically related regions. Similar to channel attention, only max-pooling operations are used in spatial attention, which can be computed as follows:

$$M_{s}\left(F\right)=\sigma \left(f^{7*7}\left(MaxPool\left(F\right)\right)\right)$$ (8)

where *f*^7*7^ represents a convolution operation with a filter size of 7×7, and σ denotes the sigmoid function.

### 3.5. QS-Attn

The QS-Attn module [20] selects the anchor q based on the significance of each feature, which was determined by its entropy. Only the most significant features that contain more domain-specific information are selected and subjected to the constraints imposed by ℒ_con_.

The attention module assigns scores to potential positions to determine similarity to all positions and to each feature. When considering a feature *F*_*x*_ ∈ ℝ^H×W×C^, the initial step involves reshaping it into a 2D matrix *Q* ∈ ℝ^HW×C^. Subsequently, this matrix is multiplied by its transposed counterpart *K* ∈ ℝ^C×HW^. Next, every row in the resulting matrix is passed through the softmax function, resulting in *A*_*g*_ ∈ ℝ^HW×HW^. The entropy Hg of Ag can be used to determine the significance of the features, as calculated using Eq (9):

$$H_{g}\left(i\right)=-\sum_{j=1}^{HW}A_{g}\left(i,j\right)\log A_{g}\left(i,j\right)$$ (9)

where *i* and *j* represent the indices of the query and key, respectively, which correspond to the row and column positions, respectively, within *A*_*g*_.

### 4. Experiments

#### 4.1. Datasets

We conducted experiments on three datasets: Cat→Dog, Horse→Zebra, and Cityscapes. The Cat↔Dog dataset contained 5,153 training images for cats and 4,739 training images for dogs, as well as 1000 validation images from the animal faces HQ (AFHQ) dataset [31]. The Horse↔Zebra dataset included 1,067 horse images and 1,344 zebra images for training and 260 test images from ImageNet [50]. The Cityscapes dataset consisted of street scenes from German cities, including 2,975 training and 1,000 validation images for each domain. The models were trained and evaluated at a resolution of 256×256.

### 4.2. Training details

To train proposed model, we mostly followed the setting of DCLGAN. Our training methodology for the generator architecture involved using a ResNet-based [51] generator based on CycleGAN [2] and CUT [19] that comprised nine residual blocks, two downsampling and upsampling blocks. Both the down-sampling and up-sampling blocks followed the pattern of two-stride convolution/deconvolution, normalization, and a rectified linear unit (ReLU). Then, residual blocks contained convolution, normalization, a ReLU, and a residual connection. To compute PatchNCE loss, features from four layers of the encoder were extracted. These four layers provided patches with resolutions of 9×9, 15×15, 35×35, and 99×99, respectively. For the first two layers, 256 random patches were extracted, whereas for the remaining two layers, CBAM and QS-Attn [20] were applied to obtain the patches. Then, the final 256-dimensional features were obtained using a 2-layer MLP (projection head H_x_, H_y_).

In our approach, we utilized a PatchGAN [4] discriminator with an architecture resembling those of CycleGAN and pix2pix [4]. In terms of how the discriminator works, it evaluates local 70 × 70 patches and assigns results to each patch. The steps of this approach can be summarized as manually cutting an image into 70×70 overlapping patches, subjecting each patch to a regular discriminator, and then computing the average results. Specifically, the discriminator receives images from each domain, passes through downsampling blocks, and generates a 30 × 30 matrix. This matrix shows the results of each element classification for the patch. Reliability is ensured by using techniques similar to CycleGAN and pix2pix, where buffers are maintained to store the last 50 images generated.

Our model used Hinge GAN loss [52], and an Adam optimizer [53] with parameters β_1_ = 0.5 and β_2_ = 0.999. Our training process include 400 epochs with a learning rate of 0.0001, which decayed linearly after the halfway point. For the generator, the ResNet-based architecture and PatchGAN was used as discriminator. Additionally, we used a batch size of 1 and instance normalization, with weights initialized using Xavier initialization [54]. During training, all images were loaded at a resolution of 286 × 286 and randomly cropped into 256×256 patches. Conversely, for testing, the images were loaded at a resolution of 256 × 256 and all the images from the test set were used for evaluation. The proposed method and existing baselines were trained on a Tesla A100-PCIE-40GB GPU using GPU driver version 450.119.04 and CUDA version 11.0.

### 4.3. Metrics

The Fréchet Inception Distance (FID) [55] is a metric widely used to evaluate the quality of generated images. Among the evaluation metrics available [56–58], we adopted the commonly used FID in the comparison experiment because most translation methods employ the FID as the quantitative measurement. It calculates the divergence between the distributions of real and generated images in deep network space and is closely related to human perception. Lower FID values suggest that generated images are more realistic and have summary statistics comparable to those of the real images in any feature space. Regarding the Cityscapes dataset, semantic segmentation was applied to the generated images using a dilated residual network (DRN) [59], and the mean average precision (mAP) metric was used to evaluate the quality of the generated images.

## 5. Results

### 5.1. Quantitative results

We quantitatively compared the performances of CUT, CycleGAN, MUNIT, DRIT, QS-Attn, DCLGAN, and the proposed method. Among these, MUNIT, DRIT, and DCLGAN are two-sided methods, whereas CUT, CycleGAN, and QS-Attn are one-sided. The experimental results are listed in Table 1, which shows that our model outperformed the other methods on the experimental datasets in terms of the FID Score. For example, for Horse→Zebra, our model showed the best FID score of 36.47. For Cityscapes, both the mAP (26.33) and FID (45.0) scores of our model were much better than those of the baselines. Our model demonstrated a superior performance not only in the original task, but also in the inverse task, as shown in Table 2.

**Table 1:** Comparison of the performances of the proposed and baseline models on the Horse→Zebra, Cat→Dog, and CityScapes datasets in terms of FID and mAP.

Method	CityScapes		Cat→Dog	Horse→Zebra	
	mAP↑	FID↓	FID↓	FID↓	sec/iter↓
CUT	24.7	56.4	76.2	45.5	0.24
QS-Attn	25.5	53.5	72.8	41.1	0.30
CycleGAN	20.4	68.6	85.9	66.8	0.40
MUNIT	16.9	91.4	104.4	133.8	0.39
DRIT	17.0	155.3	123.4	140.0	0.70
DCLGAN	22.9	49.4	60.7	43.2	0.41
**OS-DCLGAN**	**26.3**	**45.0**	**60.4**	**36.4**	**0.50**

Our model is slower than others but produces higher quality images.

**Table 2:** Comparison of the performances of the proposed and CycleGAN, CUT, and DCLGAN models on the Dog→Cat and Zebra→Horse datasets in terms of FID.

Method	Zebra→Horse	Dog→Cat
	FID↓	FID↓
CycleGAN	154.3	107.7
CUT	170.5	26.8
DCLGAN	139.5	**22.2**
**OS-DCLGAN**	**135.4**	23.1

Lower FID and higher mAP scores indicated a better performance. The highest scores are highlighted in bold; the proposed model demonstrated a competitive performance compared with those of the other methods.

### 5.2. Qualitative results

**Fig 4:**
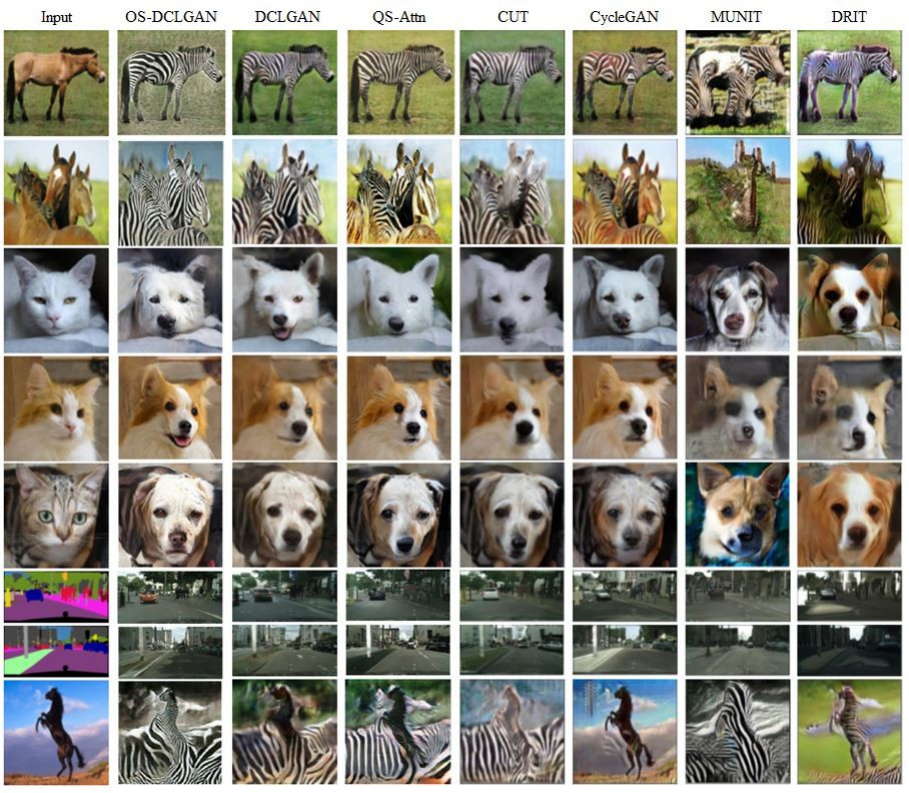
Comparison of the visual results of the proposed and baseline methods. Compared to the performance of the other methods on the Horse→Zebra, Cat→Dog, and CityScapes datasets, the performance of the proposed method is satisfactory. The last row image is an example of an uncommon pose and a failure; the model cannot distinguish the clouds and the texture of the horse.

**Fig 5:**
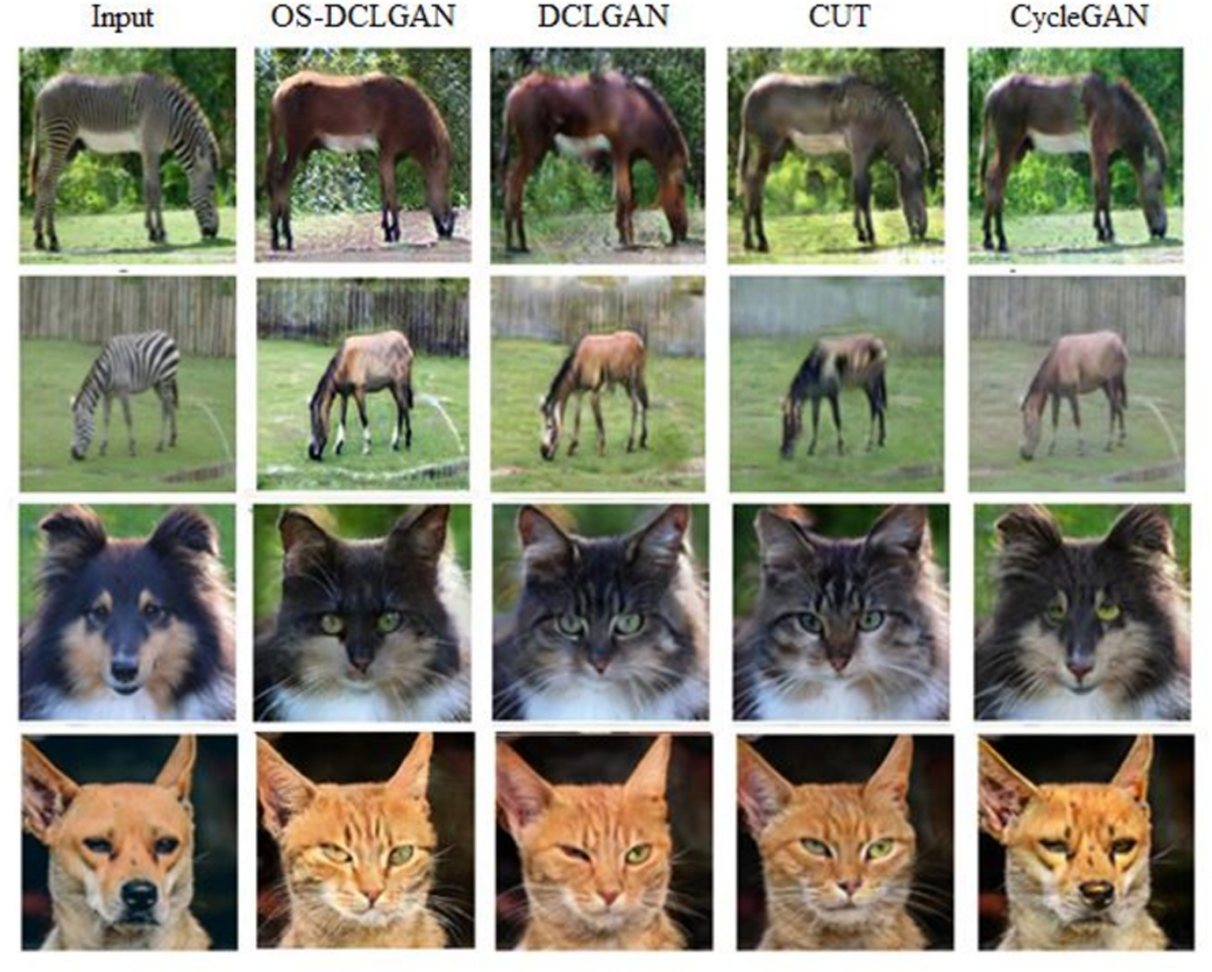
Conversions of the images of a zebra to a horse and a dog to a cat under the DCLGAN, CUT, CycleGAN, and proposed methods.

We also qualitatively compared the performance of the model with those of the baselines. Fig 4 shows the detailed visual results of the experimental datasets. Utilizing the CBAM, our model performed well, successfully generating realistic images, particularly those emphasizing important feature maps in the target domain [21]. Additionally, our model exhibited an excellent performance in terms of geometric transformation and background consistency. Fig 5 shows the visual results of the performance of different models on the Zebra→Horse and Dog→Cat datasets, confirming the ability of the proposed model to perform well in various tasks [60].

### 5.3. Ablation experiments

We experimentally demonstrated that the QS-Attn module performed better when the CBAM had been applied. We conducted ablation experiments on the Horse→Zebra dataset to analyze the isolated contributions of its components. The results of the quantitative analysis of the ablation studies are presented in Table 3.

**Table 3:** Results of the ablation study.

Ablation	Training Settings		Horse→Zebra
	Layer	Layerpooling	FID↓
(A)	2		**41.7**
(B)	4		43.2
(C)		Max	44.0
(D)		Average & Max	44.6

Layer: number of layers to which the CBAM is applied. Layerpooling: type of pooling used in the CBAM.

#### 5.3.1. Number of CBAM applied layers

The number of layers to which the CBAM was applied was varied and examined to assess the proper extent of its application. In model (A), it was applied to two of the four layers mapped to the encoder using QS-Attn, and in model (B), it was applied to all the four layers. Model (B) was slower with sec/iter 0.03 than model (A), and the result of model (B) was even worse than model (A). In addition, removing CBAM decreased training speed lightly with sec/iter 0.002, which shows the CBAM applied layers of Model (A) can learn better with acceptable speed.

#### 5.3.2. Average-pooling and max-pooling

**Fig 6:**
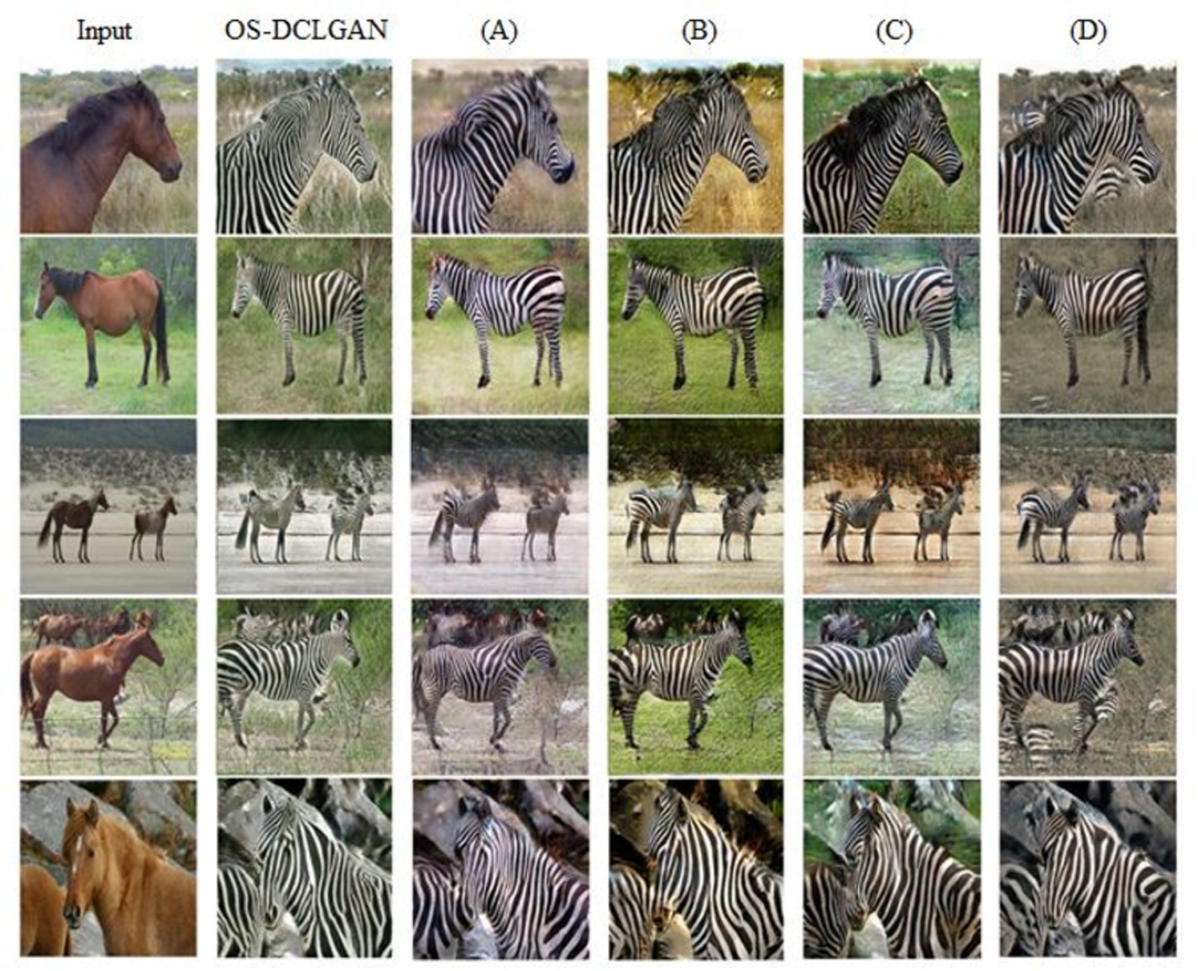
Results of the ablation studies on the Horse→Zebra dataset.

We also conducted experiments to compare the effectiveness of the following two models for the CBAM module: model (C), which used only max pooling, and model (D), which used both average and max pooling. These experiments were designed to provide insight into the optimal design of CBAM modules for image classification tasks. As shown in Table 3, the difference in performance between the method that used both average pooling and max pooling and the one that used only max pooling was not significant with the increase in the number of epochs. Fig 6 shows the visual image results of the ablation study.

## 6. Discussion

In this paper, we have proposed OS-DCLGAN to ensure stable feature representation by modifying the QS-Attn module with an inserted CBAM. Additionally, its dual learning setting enhances the training stability and enables superior feature focus, resulting in high-quality image translations. However, our approach involves a dual structure with the inclusion of the CBAM module to achieve high performance, which results in a notable increase in training time compared to those of the existing models. Therefore, OS-DCLGAN is suitable when higher accuracy and precision are expected, even though it requires more time.

**Fig 7:**
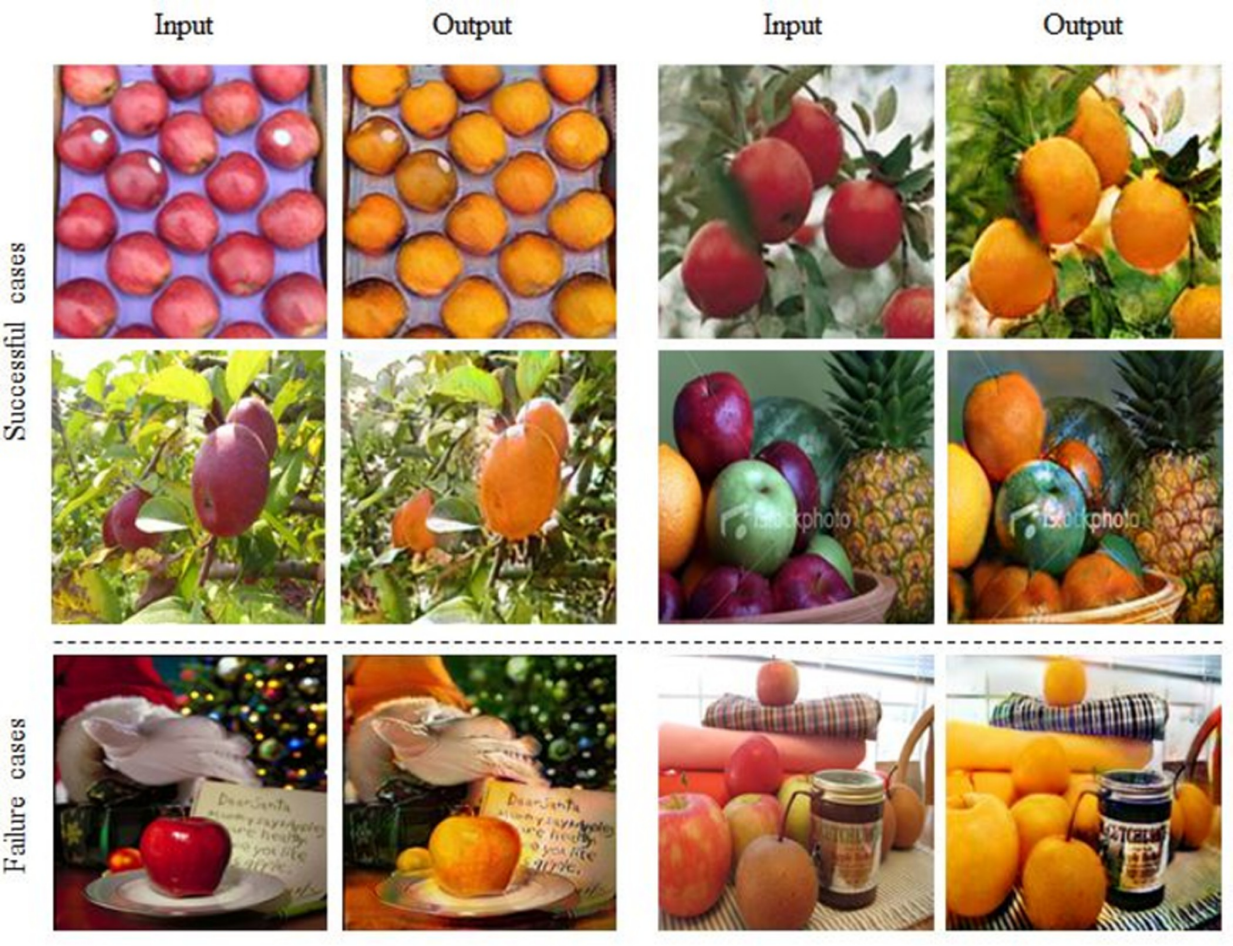
Visual translation results and some failure cases of our model between images translated from Apple→Orange dataset.

To evaluate whether our proposed model performs well even on images with complex backgrounds, we conducted additional experiments using the Apple→Orange dataset. For reproducibility, we repeated the experiments 10 times, resulting in an average FID of 102.63 with a standard deviation of 4.71. More training details regarding these experiments can be found in S3 Appendix. As shown in Fig 7, our model generally performed well on the additional dataset; however, failure occurred in some cases. We observed limitations in the achievement of geometric changes and instances in which the presence of red, the same color as an object (apple), in the background led to an orange shift. The challenge with geometric changes arises from the fact that the Apple→Orange dataset, unlike the Cat→Dog dataset, contains training data where the objects to be changed occupy smaller portions of the images.

Although OS-DCLGAN achieved the best performance in terms of FID, some issues remain that must be addressed. First, further efforts are needed to be directed towards considering reliable metrics for quantifying the performance of I2I translation models, such as the interception score and perceptual distance. Second, we could perform further research on the attention module to enable the model to capture features more accurately by exploring alternative attention models such as AttentionGAN [40], ATAGAN [61], SAGAN [62], and AGGAN [41].

The OS-DCLGAN has an architecture capable of translating between two image domains. To extend it to multi-domain tasks, an encoder that takes labels as input to generate style codes is required, and the generator needs to be modified to generate images by reflecting the style code generated by the encoder. Therefore, another interesting direction would be extending our method to multi-domain image translation by applying domain classification loss [9] and similar techniques [63–65].

Another direction for future work is to perform a comparative study by applying diffusion-based models, which are generative models that have been gaining attention recently, in an I2I task [66]. Diffusion models progressively add noise to images and then generate new data through a reverse diffusion process. Palette [67] presents conditional diffusion model for I2I translation. SRDiff [68] proposes a diffusion model for single image super resolution. The latent diffusion model (LDM) [69] improves the noise removal performance by training a diffusion model in the learned latent space. In this study, we compared GAN-based models, but as with previous work [70], future diffusion-based I2I translation methods can be compared and analyzed.

## 7. Conclusion

We propose a new framework that can outperform existing methods in terms of image extraction in the I2I field. The proposed model refines feature maps by assigning different values to points with the same entropy and extracted more meaningful point selections in the target domain. Then, it calculates the entropy of each row in the attention matrix and selects the features with the smallest N points. Additionally, the dual-wise QS-Attn module is applied to a refined feature map to increase the learning stability. The effectiveness of the proposed model was clearly shown through the experiments and ablation studies.

## Supporting information

S1 Appendix Evaluation details. (PDF)

S2 Appendix Additional results. (PDF)

S3 Appendix Apple→Orange dataset. (PDF)

## References

[1] Goodfellow I, Pouget-Abadie J, Mirza M, Xu B, Warde-Farley D, Ozair S, et al. Generative Adversarial Nets. In: Ghahramani Z, Welling M, Cortes C, Lawrence N, Weinberger KQ, editors. Advances in Neural Information Processing Systems. Curran Associates, Inc.; 2014.

[2] Zhu JY, Park T, Isola P, Efros AA. Unpaired image-to-image translation using cycle-consistent adversarial networks. In: Proceedings of the IEEE international conference on computer vision. 2017. p. 2223–32.

[3] Ledig C, Theis L, Huszár F, Caballero J, Cunningham A, Acosta A, et al. Photo-realistic single image super-resolution using a generative adversarial network. In: Proceedings of the IEEE conference on computer vision and pattern recognition. 2017. p. 4681–90.

[4] Isola P, Zhu JY, Zhou T, Efros AA. Image-to-image translation with conditional adversarial networks. In: Proceedings of the IEEE conference on computer vision and pattern recognition. 2017. p. 1125–34.

[5] Wang TC, Liu MY, Zhu JY, Tao A, Kautz J, Catanzaro B. High-resolution image synthesis and semantic manipulation with conditional gans. In: Proceedings of the IEEE conference on computer vision and pattern recognition. 2018. p. 8798–807.

[6] Park T, Liu MY, Wang TC, Zhu JY. Semantic image synthesis with spatially-adaptive normalization. In: Proceedings of the IEEE/CVF conference on computer vision and pattern recognition. 2019. p. 2337–46.

[7] Kim T, Cha M, Kim H, Lee JK, Kim J. Learning to discover cross-domain relations with generative adversarial networks. In: International conference on machine learning. PMLR; 2017. p. 1857–65.

[8] Yi Z, Zhang H, Tan P, Gong M. Dualgan: Unsupervised dual learning for image-to-image translation. In: Proceedings of the IEEE international conference on computer vision. 2017. p. 2849–57.

[9] Choi Y, Choi M, Kim M, Ha JW, Kim S, Choo J. Stargan: Unified generative adversarial networks for multi-domain image-to-image translation. In: Proceedings of the IEEE conference on computer vision and pattern recognition. 2018. p. 8789–97.

[10] Liu MY, Breuel T, Kautz J. Unsupervised image-to-image translation networks. Adv Neural Inf Process Syst. 2017;30.

[11] Mirza M, Osindero S. Conditional generative adversarial nets. ArXiv Prepr ArXiv14111784. 2014.

[12] Han J, Shoeiby M, Petersson L, Armin MA. Dual contrastive learning for unsupervised image-to-image translation. In: Proceedings of the IEEE/CVF conference on computer vision and pattern recognition. 2021. p. 746–55.

[13] Li C, Liu H, Chen C, Pu Y, Chen L, Henao R, et al. ALICE: Towards Understanding Adversarial Learning for Joint Distribution Matching. In: Guyon I, Luxburg UV, Bengio S, Wallach H, Fergus R, Vishwanathan S, et al., editors. Advances in Neural Information Processing Systems. Curran Associates, Inc.; 2017.

[14] He K, Fan H, Wu Y, Xie S, Girshick R. Momentum contrast for unsupervised visual representation learning. In: Proceedings of the IEEE/CVF conference on computer vision and pattern recognition. 2020. p. 9729–38.

[15] Chen T, Kornblith S, Norouzi M, Hinton G. A simple framework for contrastive learning of visual representations. In: International conference on machine learning. PMLR; 2020. p. 1597–607.

[16] Henaff O. Data-efficient image recognition with contrastive predictive coding. In: International conference on machine learning. PMLR; 2020. p. 4182–92.

[17] Oord A van den, Li Y, Vinyals O. Representation learning with contrastive predictive coding. ArXiv Prepr ArXiv180703748. 2018.

[18] Hjelm RD, Fedorov A, Lavoie-Marchildon S, Grewal K, Bachman P, Trischler A, et al. Learning deep representations by mutual information estimation and maximization. ArXiv Prepr ArXiv180806670. 2018.

[19] Park T, Efros AA, Zhang R, Zhu JY. Contrastive learning for unpaired image-to-image translation. In: Computer Vision–ECCV 2020: 16th European Conference, Glasgow, UK, August 23–28, 2020, Proceedings, Part IX 16. Springer; 2020. p. 319–45.

[20] Hu X, Zhou X, Huang Q, Shi Z, Sun L, Li Q. QS-Attn: Query-Selected Attention for Contrastive Learning in I2I Translation. In: Proceedings of the IEEE/CVF Conference on Computer Vision and Pattern Recognition. 2022. p. 18291–300.

[21] Woo S, Park J, Lee JY, Kweon IS. Cbam: Convolutional block attention module. In: Proceedings of the European conference on computer vision (ECCV). 2018. p. 3–19.

[22] Chen X, Duan Y, Houthooft R, Schulman J, Sutskever I, Abbeel P. Infogan: Interpretable representation learning by information maximizing generative adversarial nets. Adv Neural Inf Process Syst. 2016;29.

[23] Yan X, Yang J, Sohn K, Lee H. Attribute2image: Conditional image generation from visual attributes. In: Computer Vision–ECCV 2016: 14th European Conference, Amsterdam, The Netherlands, October 11–14, 2016, Proceedings, Part IV 14. Springer; 2016. p. 776–91.

[24] Odena A, Olah C, Shlens J. Conditional image synthesis with auxiliary classifier gans. In: International conference on machine learning. PMLR; 2017. p. 2642–51.

[25] Reed SE, Akata Z, Mohan S, Tenka S, Schiele B, Lee H. Learning what and where to draw. Adv Neural Inf Process Syst. 2016;29.

[26] Reed S, Akata Z, Yan X, Logeswaran L, Schiele B, Lee H. Generative adversarial text to image synthesis. In: International conference on machine learning. PMLR; 2016. p. 1060–9.

[27] Zhang H, Xu T, Li H, Zhang S, Wang X, Huang X, et al. Stackgan: Text to photo-realistic image synthesis with stacked generative adversarial networks. In: Proceedings of the IEEE international conference on computer vision. 2017. p. 5907–15.

[28] Dong H, Yu S, Wu C, Guo Y. Semantic image synthesis via adversarial learning. In: Proceedings of the IEEE international conference on computer vision. 2017. p. 5706–14.

[29] Huang X, Liu MY, Belongie S, Kautz J. Multimodal unsupervised image-to-image translation. In: Proceedings of the European conference on computer vision (ECCV). 2018. p. 172–89.

[30] Benaim S, Wolf L. One-sided unsupervised domain mapping. Adv Neural Inf Process Syst. 2017;30.

[31] Choi Y, Uh Y, Yoo J, Ha JW. Stargan v2: Diverse image synthesis for multiple domains. In: Proceedings of the IEEE/CVF conference on computer vision and pattern recognition. 2020. p. 8188–97.

[32] Zhao Y, Wu R, Dong H. Unpaired image-to-image translation using adversarial consistency loss. In: Computer Vision–ECCV 2020: 16th European Conference, Glasgow, UK, August 23–28, 2020, Proceedings, Part IX 16. Springer; 2020. p. 800–15.

[33] Lee HY, Tseng HY, Huang JB, Singh M, Yang MH. Diverse image-to-image translation via disentangled representations. In: Proceedings of the European conference on computer vision (ECCV). 2018. p. 35–51.

[34] Lee HY, Tseng HY, Mao Q, Huang JB, Lu YD, Singh M, et al. Drit++: Diverse image-to-image translation via disentangled representations. Int J Comput Vis. 2020;128:2402–17.

[35] Richardson E, Alaluf Y, Patashnik O, Nitzan Y, Azar Y, Shapiro S, et al. Encoding in style: a stylegan encoder for image-to-image translation. In: Proceedings of the IEEE/CVF conference on computer vision and pattern recognition. 2021. p. 2287–96.

[36] Zhu JY, Zhang R, Pathak D, Darrell T, Efros AA, Wang O, et al. Toward multimodal image-to-image translation. Adv Neural Inf Process Syst. 2017;30.

[37] Liu MY, Huang X, Mallya A, Karras T, Aila T, Lehtinen J, et al. Few-shot unsupervised image-to-image translation. In: Proceedings of the IEEE/CVF international conference on computer vision. 2019. p. 10551–60.

[38] Xu T, Zhang P, Huang Q, Zhang H, Gan Z, Huang X, et al. Attngan: Fine-grained text to image generation with attentional generative adversarial networks. In: Proceedings of the IEEE conference on computer vision and pattern recognition. 2018. p. 1316–24.

[39] Alami Mejjati Y, Richardt C, Tompkin J, Cosker D, Kim KI. Unsupervised attention-guided image-to-image translation. Adv Neural Inf Process Syst. 2018;31.

[40] Chen X, Xu C, Yang X, Tao D. Attention-gan for object transfiguration in wild images. In: Proceedings of the European conference on computer vision (ECCV). 2018. p. 164–80.

[41] Tang H, Xu D, Sebe N, Yan Y. Attention-guided generative adversarial networks for unsupervised image-to-image translation. In: 2019 International Joint Conference on Neural Networks (IJCNN). IEEE; 2019. p. 1–8.

[42] Yang S, Sun M, Lou X, Yang H, Zhou H. An unpaired thermal infrared image translation method using GMA-CycleGAN. Remote Sens. 2023;15(3):663.

[43] Nizan O, Tal A. Breaking the cycle-colleagues are all you need. In: Proceedings of the IEEE/CVF conference on computer vision and pattern recognition. 2020. p. 7860–9.

[44] Pumarola A, Agudo A, Martinez AM, Sanfeliu A, Moreno-Noguer F. Ganimation: Anatomically-aware facial animation from a single image. In: Proceedings of the European conference on computer vision (ECCV). 2018. p. 818–33. doi: 10.1007/978-3-030-01249-6_50 30465044 PMC6240441

[45] Zheng C, Cham TJ, Cai J. The spatially-correlative loss for various image translation tasks. In: Proceedings of the IEEE/CVF conference on computer vision and pattern recognition. 2021. p. 16407–17.

[46] Wu H, Qu Y, Lin S, Zhou J, Qiao R, Zhang Z, et al. Contrastive learning for compact single image dehazing. In: Proceedings of the IEEE/CVF Conference on Computer Vision and Pattern Recognition. 2021. p. 10551–60.

[47] Zhang Y, Tang F, Dong W, Huang H, Ma C, Lee TY, et al. Domain enhanced arbitrary image style transfer via contrastive learning. In: ACM SIGGRAPH 2022 Conference Proceedings. 2022. p. 1–8.

[48] Luo X, Ju W, Qu M, Chen C, Deng M, Hua XS, et al. Dualgraph: Improving semi-supervised graph classification via dual contrastive learning. In: 2022 IEEE 38th International Conference on Data Engineering (ICDE). IEEE; 2022. p. 699–712.

[49] Ju W, Gu Y, Luo X, Wang Y, Yuan H, Zhong H, et al. Unsupervised graph-level representation learning with hierarchical contrasts. Neural Netw. 2023;158:359–68. doi: 10.1016/j.neunet.2022.11.019 36516542

[50] Deng J, Dong W, Socher R, Li LJ, Li K, Fei-Fei L. Imagenet: A large-scale hierarchical image database. In: 2009 IEEE conference on computer vision and pattern recognition. Ieee; 2009. p. 248–55.

[51] He K, Zhang X, Ren S, Sun J. Deep residual learning for image recognition. In: Proceedings of the IEEE conference on computer vision and pattern recognition. 2016. p. 770–8.

[52] Lim JH, Ye JC. Geometric gan. ArXiv Prepr ArXiv170502894. 2017.

[53] Kingma DP, Ba J. Adam: A method for stochastic optimization. ArXiv Prepr ArXiv14126980. 2014.

[54] Glorot X, Bengio Y. Understanding the difficulty of training deep feedforward neural networks. In: Proceedings of the thirteenth international conference on artificial intelligence and statistics. JMLR Workshop and Conference Proceedings; 2010. p. 249–56.

[55] Heusel M, Ramsauer H, Unterthiner T, Nessler B, Hochreiter S. Gans trained by a two time-scale update rule converge to a local nash equilibrium. Adv Neural Inf Process Syst. 2017;30.

[56] Salimans T, Goodfellow I, Zaremba W, Cheung V, Radford A, Chen X. Improved techniques for training gans. Adv Neural Inf Process Syst. 2016;29.

[57] Dosovitskiy A, Brox T. Generating images with perceptual similarity metrics based on deep networks. Adv Neural Inf Process Syst. 2016;29.

[58] Zhang R, Isola P, Efros AA, Shechtman E, Wang O. The unreasonable effectiveness of deep features as a perceptual metric. In: Proceedings of the IEEE conference on computer vision and pattern recognition. 2018. p. 586–95.

[59] Yu F, Koltun V, Funkhouser T. Dilated residual networks. In: Proceedings of the IEEE conference on computer vision and pattern recognition. 2017. p. 472–80.

[60] Zhang R, Isola P, Efros AA. Colorful image colorization. In: Computer Vision–ECCV 2016: 14th European Conference, Amsterdam, The Netherlands, October 11–14, 2016, Proceedings, Part III 14. Springer; 2016. p. 649–66.

[61] Kastaniotis D, Ntinou I, Tsourounis D, Economou G, Fotopoulos S. Attention-aware generative adversarial networks (ATA-GANs). In: 2018 IEEE 13th Image, Video, and Multidimensional Signal Processing Workshop (IVMSP). IEEE; 2018. p. 1–5.

[62] Zhang H, Goodfellow I, Metaxas D, Odena A. Self-attention generative adversarial networks. In: International conference on machine learning. PMLR; 2019. p. 7354–63.

[63] Yu X, Cai X, Ying Z, Li T, Li G. Singlegan: Image-to-image translation by a single-generator network using multiple generative adversarial learning. In: Computer Vision–ACCV 2018: 14th Asian Conference on Computer Vision, Perth, Australia, December 2–6, 2018, Revised Selected Papers, Part V 14. Springer; 2019. p. 341–56.

[64] Huang S, He C, Cheng R. SoloGAN: Multi-domain Multimodal Unpaired Image-to-Image Translation via a Single Generative Adversarial Network. IEEE Trans Artif Intell. 2022;3(5):722–37.

[65] Jeong S, Lee J, Sohn K. Multi-domain unsupervised image-to-image translation with appearance adaptive convolution. In: ICASSP 2022–2022 IEEE International Conference on Acoustics, Speech and Signal Processing (ICASSP). IEEE; 2022. p. 1750–4.

[66] Ho J, Jain A, Abbeel P. Denoising diffusion probabilistic models. Adv Neural Inf Process Syst. 2020;33:6840–51.

[67] Saharia C, Chan W, Chang H, Lee C, Ho J, Salimans T, et al. Palette: Image-to-image diffusion models. In: ACM SIGGRAPH 2022 Conference Proceedings. 2022. p. 1–10.

[68] Li H, Yang Y, Chang M, Chen S, Feng H, Xu Z, et al. Srdiff: Single image super-resolution with diffusion probabilistic models. Neurocomputing. 2022;479:47–59.

[69] Rombach R, Blattmann A, Lorenz D, Esser P, Ommer B. High-resolution image synthesis with latent diffusion models. In: Proceedings of the IEEE/CVF conference on computer vision and pattern recognition. 2022. p. 10684–95.

[70] Li L, Ma L. Injecting-Diffusion: Inject Domain-Independent Contents into Diffusion Models for Unpaired Image-to-Image Translation. In: 2023 IEEE International Conference on Multimedia and Expo (ICME). IEEE; 2023. p. 282–7.

