# Supplementary material for: Object-stable unsupervised dual contrastive learning image-to-image translation with query-selected attention and convolutional block attention module
Source: PLoS One. 2023 Nov 6;18(11):e0293885. doi: 10.1371/journal.pone.0293885 (PMC10627467; doi:10.1371/journal.pone.0293885)
Supplement: S1 Appendix — (PDF) [file pone.0293885.s001.pdf]

S1 Appendix. Evaluation details

To compute the Fréchet Inception Distance (FID) (1) score on the test set images, we calculate the mean and variance distance of the generated and real images in a deep feature space. To achieve this, we first resize the images to 299x299 and then extract deep features from them using a pretrained Inception model. We used the default setting of https://github.com/mseitzer/pytorch-fid for this purpose.

For evaluating mAP, the semantic segmentation metrics on the Cityscapes dataset, we employed a pretrained DRN-D-22 (2) model to segment the generated images and compared the results with the corresponding ground truth labels. The pretrained DRN-D-22 model was trained using a batch size of 32 and a learning rate of 0.01 for 250 epochs at a resolution of 256x128, and can be found at https://github.com/WeilunWang/NEGCUT. We used bicubic downsampling to resize the input images to 256x128 before passing them to the segmentation model, and down-sampled the ground truth labels to the same size using nearest-neighbor sampling. The original training code for DRN-D-22 can be accessed at https://github.com/fyu/drn.

References

1. Heusel M, Ramsauer H, Unterthiner T, Nessler B, Hochreiter S. Gans trained by a two time-scale update rule converge to a local nash equilibrium. Adv Neural Inf Process Syst. 2017;30.
2. Yu F, Koltun V, Funkhouser T. Dilated residual networks. In: Proceedings of the IEEE conference on computer vision and pattern recognition. 2017. p. 472–80.
