# Supplementary material for: Object-stable unsupervised dual contrastive learning image-to-image translation with query-selected attention and convolutional block attention module
Source: PLoS One. 2023 Nov 6;18(11):e0293885. doi: 10.1371/journal.pone.0293885 (PMC10627467; doi:10.1371/journal.pone.0293885)
Supplement: S2 Appendix — (PDF) [file pone.0293885.s002.pdf]

S2 Appendix. Additional results

In addition to the images we compared in the main paper, we can see additional qualitative results. Fig 1. shows the results for Horse ↔ Zebra, Fig 2. for Cat ↔ Dog, and Fig 3. for cityscapes. Overall, our proposed model outperformed the other models in terms of geometric change and background-consistency for most tasks.

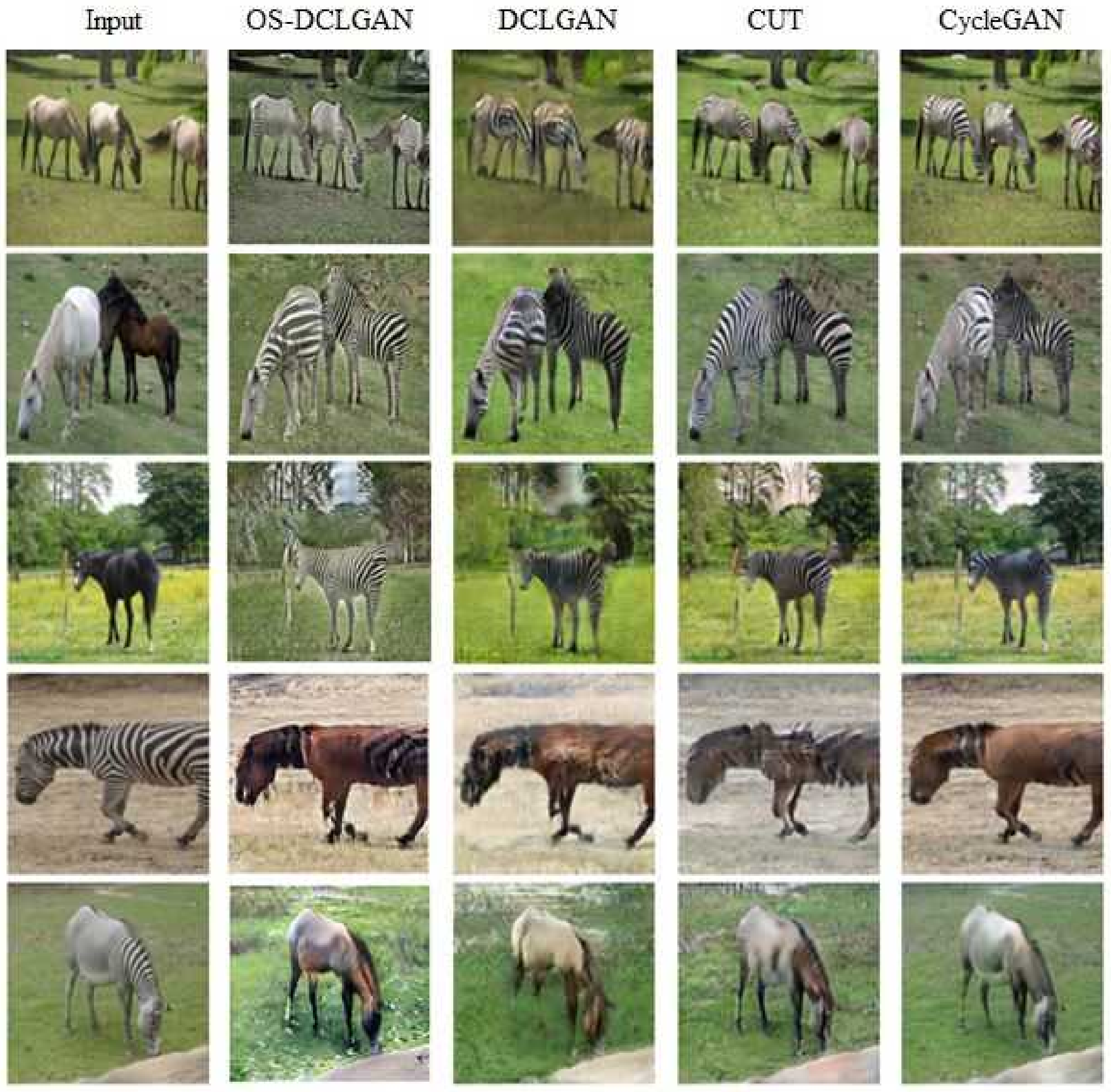

Fig 1. Additional results of Horse↔Zebra.

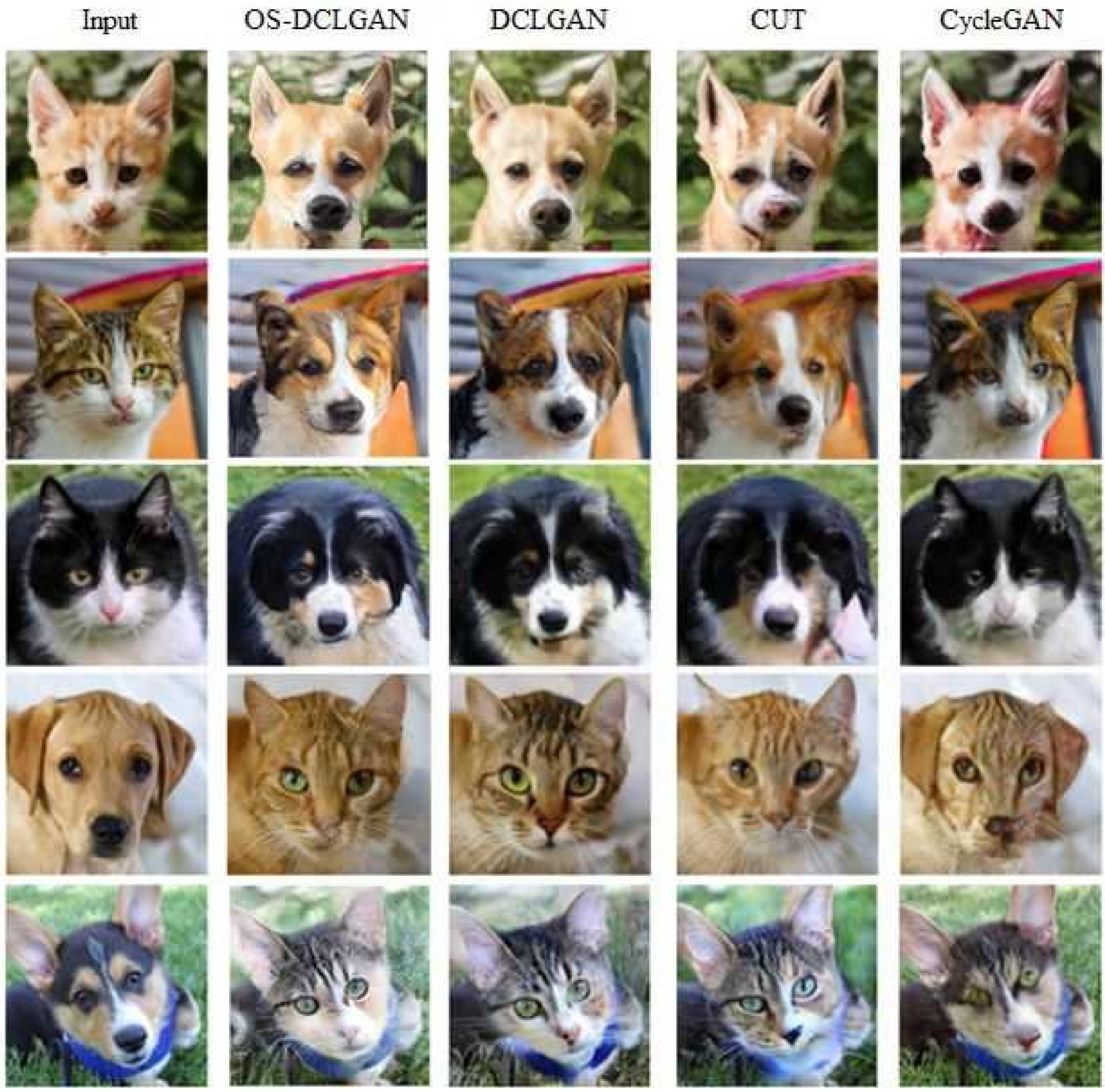

Fig 2. Additional results of Cat↔Dog.

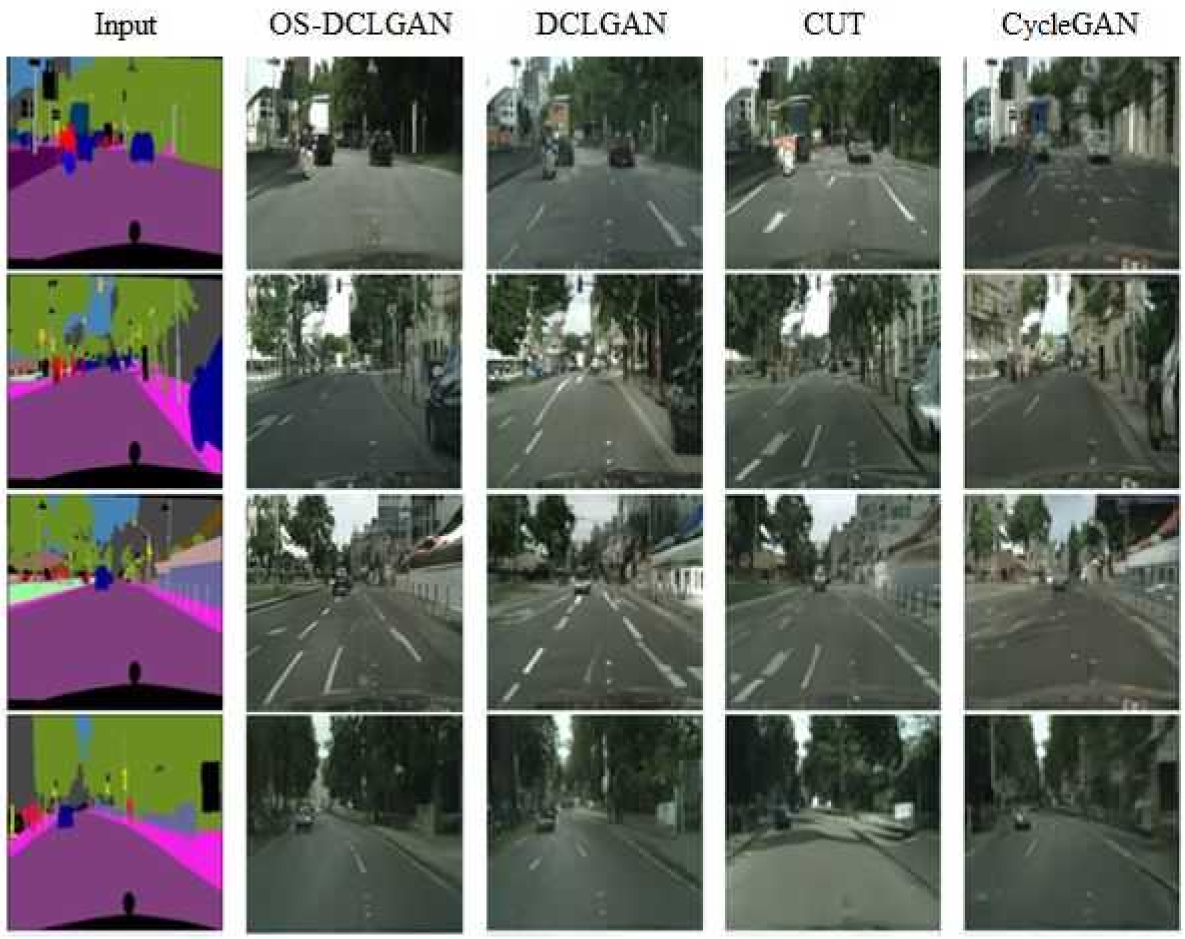

Fig 3. Additional results of CityScapes.
