# Supplementary material for: Object-stable unsupervised dual contrastive learning image-to-image translation with query-selected attention and convolutional block attention module
Source: PLoS One. 2023 Nov 6;18(11):e0293885. doi: 10.1371/journal.pone.0293885 (PMC10627467; doi:10.1371/journal.pone.0293885)
Supplement: S3 Appendix — (PDF) [file pone.0293885.s003.pdf]

S3 Appendix. Apple→Orange dataset

We conducted additional experiments using the Apple→Orange dataset. It contains 1019 orange images and 995 apple images in the trainingset from ImageNet (1). For testing, we use 266 orange images. The experimental conditions and evaluation metrics remain consistent with the training details outlined in Chapter 4 of the paper. The results of this experiment regarding training time can be found in Table 1. Our proposed model falls on the slower side compared to other state-of-the-art (SOTA) models.

Table 1. Training time (in hours) per model on the Apple→Orange dataset. The Apple→Orange dataset contains about 1,000 images and used a Tesla A100-PCIE-40GB GPU.

	Apple→Orange
Method	Training time(*h*) ↓
QS-Attn	**12**
DCLGAN	18
**OS-DCLGAN**	20

References

1. Deng J, Dong W, Socher R, Li LJ, Li K, Fei-Fei L. Imagenet: A large-scale hierarchical image database. In: 2009 IEEE conference on computer vision and pattern recognition. Ieee; 2009. p. 248–55.
